# Simple approaches to characterising multiple long‐term conditions (multimorbidity) and rates of emergency hospital admission: Findings from 495,465 UK Biobank participants

**DOI:** 10.1111/joim.13567

**Published:** 2022-09-21

**Authors:** Richard M. Dodds, Jonathan G. Bunn, Susan J. Hillman, Antoneta Granic, James Murray, Miles D. Witham, Sian M. Robinson, Rachel Cooper, Avan A. Sayer

**Affiliations:** ^1^ AGE Research Group Newcastle University Institute for Translational and Clinical Research Newcastle upon Tyne UK; ^2^ NIHR Newcastle Biomedical Research Centre Newcastle University and Newcastle upon Tyne NHS Foundation Trust Newcastle upon Tyne UK; ^3^ Department of Sport and Exercise Sciences Musculoskeletal Science and Sports Medicine Research Centre Manchester Metropolitan University Manchester UK

**Keywords:** death, emergency hospital admission, multimorbidity, multiple long‐term conditions

## Abstract

**Background:**

Numerous approaches are used to characterise multiple long‐term conditions (MLTC), including counts and indices. Few studies have compared approaches within the same dataset. We aimed to characterise MLTC using simple approaches, and compare their prevalence estimates of MLTC and associations with emergency hospital admission in the UK Biobank.

**Methods:**

We used baseline data from 495,465 participants (age 38–73 years) to characterise MLTC using four approaches: Charlson index (CI), Byles index (BI), count of 43 conditions (CC) and count of body systems affected (BC). We defined MLTC as more than two conditions using CI, BI and CC, and more than two body systems using BC. We categorised scores (incorporating weightings for the indices) from each approach as 0, 1, 2 and 3+. We used linked hospital episode statistics and performed survival analyses to test associations with an endpoint of emergency hospital admission or death over 5 years.

**Results:**

The prevalence of MLTC was 44% (BC), 33% (CC), 6% (BI) and 2% (CI). Higher scores using all approaches were associated with greater outcome rates independent of sex and age group. For example, using CC, compared with score 0, score 2 had 1.95 (95% CI: 1.91, 1.99) and a score of 3+ had 3.12 (95% CI: 3.06, 3.18) times greater outcome rates. The discriminant value of all approaches was modest (C‐statistics 0.60–0.63).

**Conclusions:**

The counts classified a greater proportion as having MLTC than the indices, highlighting that prevalence estimates of MLTC vary depending on the approach. All approaches had strong statistical associations with emergency hospital admission but a modest ability to identify individuals at risk.

## Graphical abstract

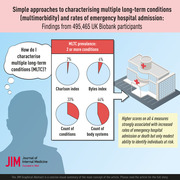

## Introduction

Multiple long‐term conditions (MLTC), also known as multimorbidity, describes the coexistence of two or more long‐term conditions in the same individual, and approximately one quarter of the population in high‐income countries such as the UK live with MLTC [1]. People living with MLTC are the main recipients of health and social care services [2], and MLTC is linked to a wide range of adverse consequences including reduced quality of life and loss of physical independence [3]. Compared to many individual long‐term conditions, there is less evidence regarding the prevention, diagnosis and treatment of MLTC and this is now a major focus of research [4].

The characterisation of MLTC is important for several reasons, including to establish the presence or absence of MLTC and hence compare the prevalence between different groups. The varied nature of MLTC means that it is important to be able to describe its complexity, including the number of conditions present and the number of organ systems affected. Many different approaches have been used to characterise MLTC [5], with no consensus to date on which method(s) should be used to describe MLTC as required to ensure a greater degree of comparability and reproducibility across studies. A recent goal has been to use methods to group individuals or conditions into specific clusters [6, 7], for example, those who tend to experience certain healthcare outcomes, or to identify aetiological factors including genetic variants that may predispose to specific combinations of LTC.

A well‐established strategy to characterising MLTC is the use of counts or indices (counts where individual conditions are given weightings) [8]. Counts and indices can be used as exposures in epidemiological analyses of the relationship with healthcare outcomes. Emergency hospital admission presents a significant burden both to individuals and the healthcare system, and the risk is known to be greater amongst those with higher scores on MLTC counts and indices assessed using routine healthcare data [9]. There is increasing interest in characterising MLTC in cohort studies, including the UK Biobank [10, 11]. Benefits of doing this include the opportunity to assess individuals at an earlier stage of MLTC development, which may yield insights for prevention, and to take advantage of measures not typically available in routine data sources including genotyping. Our aims using data from the UK Biobank Study were to compare the prevalence of MLTC characterised using two count and two index approaches, and to test the associations between these different characterisations of MLTC and emergency hospital admission over a 5‐year period.

## Methods

### Source of data and variables used

UK Biobank is a large prospective epidemiological study designed to investigate the roles of genetic, lifestyle and environmental factors in health and disease in mid‐ to later life [12]. In summary, 502,412 participants aged 37–73 years were recruited and seen for baseline assessment at 22 centres in England, Wales and Scotland between 2006 and 2010. Ethical approval for the UK Biobank was obtained from the North West Multi‐Centre Research Ethics Committee and participants provided written informed consent. This ethical approval covers the analysis of all data in the present study, including linkage to participants’ health‐related records. We included participants with information from the baseline assessment on MLTC and covariates, and linked records for the combined endpoint of emergency hospital admission or death in the 5 years that followed.

#### Assessment and characterisation of MLTC

At their baseline assessment, participants in the UK Biobank study were asked whether they had ever been told by a doctor that they had: a heart attack, angina, stroke, high blood pressure, blood clot in the leg, blood clot in the lung, emphysema/chronic bronchitis, asthma, diabetes, cancer or any other serious medical conditions. Those who responded yes to any one of these questions were then asked to complete an interview in which a research nurse recorded details of all long‐term conditions against a hierarchical tree of over 450 conditions similar to that used in the International Classification of Diseases‐10 (ICD‐10) classification [13].

We implemented two index (a count of specific conditions with a weighting assigned to each) and two count‐based approaches to characterising MLTC. Our selection of indices was based on findings from a recent systematic review that recommended examining existing indices rather than creating new ones [8]. Of the 35 existing indices identified by the review authors, we selected a priori to use two: (i) the Charlson index (CI), which includes 19 different conditions, is widely known and has been commonly used to characterise multimorbidity for many years [14] and (ii) the Byles index (BI), which includes nine self‐reported long‐term conditions, has previously been used to predict our main outcome of interest (i.e., risk of hospital admission) [15] and was one of the few indices classified as high quality by systematic review authors [8]. The two count‐based approaches drew on different lists of conditions. The Gallacher count includes 43 condition groupings as previously implemented in the UK Biobank [16] (henceforth CC, condition count). The Dodds count reports the number of body systems in which a person has one or more long‐term conditions [17], drawn from a list similar to that used in the ICD‐10 classification (henceforth BC, body system count). For further details on the specific items used in each of the four approaches, see Supporting Information—Methods 1 and 2. The scores from the approaches were all categorised as 0, 1, 2 and 3 or greater. We also used the presence of two or more items to assess the prevalence of MLTC using each approach.

#### Assessment of covariates

Covariates that are widely available in population‐based studies, assessed during primary care health checks and may potentially confound the main associations of interest were selected a priori. These factors were all assessed during the baseline assessment. We described socioeconomic position using the Townsend deprivation index. This is a commonly used area‐based measure of socioeconomic position derived from postcodes, which was categorised into fifths from the least deprived to the most deprived. We calculated body mass index (BMI) using measured height and weight, categorised as normal (<25 kg/m^2^), overweight (≥25 and <30 kg/m^2^) and obese (≥30 kg/m^2^). We categorised self‐reported smoking status and alcohol status as never, previous and current.

#### Assessment of emergency hospital admission (or death)

Participants were linked to National Health Service (NHS) central registers for information on death and to country‐specific hospital admission records (Hospital Episode Statistics, Scottish Morbidity Records and the Patient Episode Database for Wales) [18]. To minimise error, participants were linked using their 10‐digit NHS number, postcode, sex and age. Notifications of deaths were recorded in the UK Biobank via regular updates from NHS Digital for participants in England and Wales and from the NHS Central Register for participants in Scotland. We calculated time to event in days from the date of baseline assessment to whichever of emergency hospital admission or death occurred first over 5 years of follow‐up, which was taken as 1826 days. Participants who withdrew consent for future linkage of data during this time were censored at the interval between baseline assessment and the date recorded in the UK Biobank as lost to follow‐up. Participants not experiencing emergency admission or death were censored at 1826 days.

### Statistical analyses

We excluded those with missing data on MLTC (*n* = 862, 0.2% of the original sample) and then excluded those with missing data on covariates (*n* = 6085, 1.2% of the original sample), leaving an analysis sample of *n* = 495,465. We produced descriptive statistics for the MLTC approaches and covariates. We compared participants with and without the combined endpoint, testing for differences between continuous variables using the Wilcoxon rank sum test and categorical variables using the chi‐squared test.

We used Kaplan–Meier plots to visualise the relationships between each MLTC approach and the combined endpoint. We inspected plots of the Nelson–Aalen cumulative hazard estimates to check that there were no violations of the proportional hazards assumption. We then performed Cox regression analyses for each MLTC approach, using two models: (i) adjusted for age band (38–44, 45–54, 55–64 and 65–74 years) and sex and (ii) also adjusted for Townsend deprivation index, BMI category and smoking and alcohol status. We used Harrell's C‐statistic to compare the performance of each MLTC approach in the prediction of the combined endpoint in models adjusted for age and sex only and models adjusted for all covariates. We found evidence of interactions by sex and age, so we repeated our Cox regression analyses stratified by sex and age group (38–54 compared with 55–74 years). As sensitivity analyses, we reran the main models using death as the sole outcome and reran analyses of the CI using updated weights [19, 20]. We undertook all analyses in R version 4.1.2 [21].

## Results

### Prevalence of MLTC using four different approaches

**Fig. 1:**
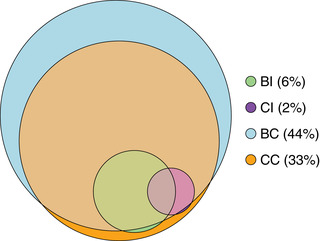
Venn diagram showing the overlap between four different approaches to characterising multiple long‐term conditions. BC, body system count; BI, Byles index; CC, condition count; CI, Charlson index. Percentages shown indicate the proportion of participants with two or more conditions according to the criteria for each of the four approaches.

The sample had a median age of 58 [interquartile range (q25, q75) 50, 63] years at baseline and 54.5% were female. Most participants had a score of 1 or greater using the two count approaches (BC: 75.6% and CC: 65.8%), compared with a minority of participants using the two index approaches (BI: 29.1% and CI: 17.1%). We observed the same ranking of the different approaches when used to assess the prevalence of MLTC (the presence of two or more items in each approach), in descending order of prevalence: BC (44.0%), CC (33.0%), BI (5.7%) and CI (1.9%). There was a high degree of overlap between the different approaches, with the less prevalent approaches nested within the more prevalent ones, as shown in Fig. 1. For example, of those with MLTC assessed using CC, 95.8% also had MLTC assessed using the more prevalent BC approach.

### Associations with emergency hospital admission (or death)

A total of 94,047 participants (19.0%) had at least one emergency hospital admission and/or died within the first 5 years after baseline assessment. As shown in Table 1, those with this combined endpoint were older and more likely to be male, to live in an area of greater socioeconomic deprivation, to be obese and ever smokers, and less likely to be a current alcohol drinker. Higher scores in all four MLTC approaches had statistically significant associations with greater risk of emergency hospital admission (or death) as a binary variable, as also shown in Table 1.

**Table 1:** Baseline characteristics of the UK Biobank analytic sample, by study outcome

Characteristic*		Not admitted and alive	Admitted or died	Lost to follow‐up
Age (years) at baseline (median, interquartile range q25, q75)		57 (50, 63)	60 (53, 65)	61 (52.5, 65)
Age (years) at baseline	38–44	44,062 (10.98)	6909 (7.35)	2 (1.96)
category (*N* [%])	45–54	118,684 (29.57)	21,766 (23.14)	26 (25.49)
	55–64	168,911 (42.09)	40,541 (43.11)	46 (45.10)
	65–74	69,659 (17.36)	24,831 (26.40)	28 (27.45)
Sex (*N* [%])	Female	223,144 (55.60)	46,637 (49.59)	68 (66.67)
	Male	178,172 (44.40)	47,410 (50.41)	34 (33.33)
Townsend deprivation index	1 (least deprived)	83,464 (20.80)	16,194 (17.22)	24 (23.53)
fifths (*N* [%])	2	82,250 (20.50)	17,010 (18.09)	20 (19.61)
	3	81,280 (20.25)	18,072 (19.22)	24 (23.53)
	4	79,803 (19.89)	19,253 (20.47)	14 (13.73)
	5 (most deprived)	74,519 (18.57)	23,518 (25.01)	20 (19.61)
BMI (kg/m^2^) (mean [standard deviation])		27.2 (4.64)	28.4 (5.32)	27.6 (5.22)
BMI category (kg/m^2^)	≤25	138,608 (34.54)	25,273 (26.87)	36 (35.29)
(*N* [%])	25–30	171,477 (42.73)	38,963 (41.43)	39 (38.24)
	>30	91,231 (22.73)	29,811 (31.70)	27 (26.47)
Smoking status (*N* [%])	Never	225,899 (56.29)	45,302 (48.17)	63 (61.76)
	Previous	136,385 (33.98)	35,509 (37.76)	32 (31.37)
	Current	39,032 (9.73)	13,236 (14.07)	7 (6.86)
Alcohol use (*N* [%])	Current	372,194 (92.74)	83,573 (88.86)	90 (88.24)
	Never	16,546 (4.12)	5317 (5.65)	10 (9.8)
	Previous	12,576 (3.13)	5157 (5.48)	2 (1.96)
CI score (*N* [%])	0	342,977 (85.46)	67,911 (72.21)	74 (72.55)
	1	25,564 (6.37)	12,386 (13.17)	10 (9.80)
	2	29,748 (7.41)	11,137 (11.84)	16 (15.69)
	3+	3027 (0.75)	2613 (2.78)	2 (1.96)
BI score (*N* [%])	0	297,207 (74.06)	54,178 (57.61)	61 (59.80)
	1	63,399 (15.8)	21,401 (22.76)	22 (21.57)
	2	32,331 (8.06)	12,937 (13.76)	16 (15.69)
	3+	8379 (2.09)	5531 (5.88)	3 (2.94)
CC score (*N* [%])	0	149,170 (37.17)	20,291 (21.58)	34 (33.33)
	1	134,510 (33.52)	27,878 (29.64)	33 (32.35)
	2	72,632 (18.1)	21,967 (23.36)	16 (15.69)
	3+	45,004 (11.21)	23,911 (25.42)	19 (18.63)
BC score (*N* [%])	0	107,386 (26.76)	13,505 (14.36)	20 (19.61)
	1	130,924 (32.62)	25,522 (27.14)	32 (31.37)
	2	90,087 (22.45)	24,692 (26.25)	27 (26.47)
	3+	72,919 (18.17)	30,328 (32.25)	23 (22.55)
Proportion with multiple long‐term conditions (*N* [%])^a^	CI	4760 (1.19)	4422 (4.7)	4 (3.92)
	BI	17,025 (4.24)	11,097 (11.80)	5 (4.90)
	CC	117,636 (29.31)	45,878 (48.78)	35 (34.31)
	BC	163,006 (40.62)	55,020 (58.50)	50 (49.02)

*Note: N* = 495,465.

Abbreviations: BC, body system count; BI, Byles index; BMI, body mass index; CC, condition count; CI, Charlson index.

^a^ Defined as having two or more items in each approach.

* *P*‐value for differences between participants compare those with and without admission (or death) all <0.001, analysed using chi‐squared tests for categorical variables and Wilcoxon rank sum tests for continuous variables.

**Fig. 2:**
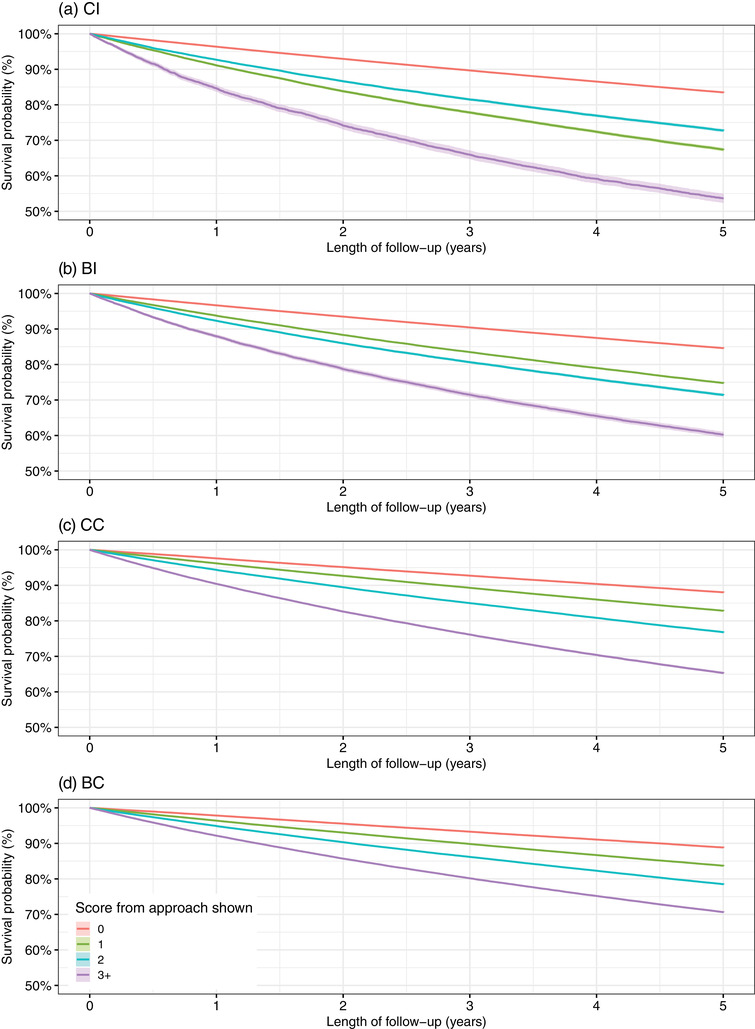
Kaplan–Meier survival probabilities (and 95% confidence intervals) for emergency hospital admission (or death) stratified by scores from each of the four approaches used to characterise multiple long‐term conditions. BC, body system count; BI, Byles index; CC, condition count; CI, Charlson index.

All four approaches showed graded relationships between a higher score and lower survival (i.e., a greater probability of emergency hospital admission or death), as shown in the Kaplan–Meier curves in Fig. 2. The one exception to this pattern was with the CI approach (Fig. 2a), where those with a score of 2 had increased survival compared with those with a score of 1. As shown in Table 2, all four approaches remained associated with emergency hospital admission (or death) when included in Cox regression models along with age and sex. The associations remained albeit attenuated when other covariates were included. The regression models also confirmed that individuals with a CI approach score of 2 had significantly lower outcome rates than those with a score of 1. All four approaches made modest increases in the prediction of emergency hospital admission (or death). For example, the C‐statistic for a model with age and sex was 0.57 (95% CI: 0.569–0.572) and this increased slightly to 0.63 (95% CI: 0.628–0.632) when the score from the CC approach was added to the model.

**Table 2:** Findings from Cox regression models for the relationship between each approach to characterising multiple long‐term conditions (MLTC) and rates of emergency hospital admission (or death)

	Approach to characterising MLTC				
Covariates in model	None	CI	BI	CC	BC
Age and sex					
Hazard ratio (95% CI)					
Score 0	n/a	1.00 (ref.)	1.00 (ref.)	1.00 (ref.)	1.00 (ref.)
Score 1	n/a	2.00 (1.96–2.04)	1.65 (1.63–1.68)	1.43 (1.40–1.45)	1.44 (1.41–1.47)
Score 2	n/a	1.67 (1.64–1.71)	1.91 (1.87–1.94)	1.95 (1.91–1.99)	1.91 (1.87–1.95)
Score 3+	n/a	3.12 (3.00–3.24)	2.79 (2.71–2.86)	3.12 (3.06–3.18)	2.73 (2.68–2.79)
C‐statistic (95% CI)	0.57 (0.569–0.572)	0.601 (0.599–0.603)	0.611 (0.608–0.612)	0.630 (0.628–0.632)	0.621 (0.619–0.623)
Multivariable model^a^					
Hazard ratio (95% CI)					
Score 0	n/a	1.00 (ref.)	1.00 (ref.)	1.00 (ref.)	1.00 (ref.)
Score 1	n/a	1.82 (1.78–1.85)	1.51 (1.48–1.53)	1.38 (1.35–1.41)	1.39 (1.36–1.42)
Score 2	n/a	1.64 (1.60–1.67)	1.79 (1.75–1.82)	1.82 (1.78–1.85)	1.79 (1.75–1.83)
Score 3+	n/a	2.75 (2.64–2.86)	2.41 (2.35–2.48)	2.73 (2.67–2.78)	2.44 (2.39–2.49)
C‐statistic (95% CI)	0.61 (0.609–0.613)	0.629 (0.627–0.630)	0.631 (0.629–0.633)	0.643 (0.642–0.645)	0.638 (0.636–0.640)

*Note: P*‐values comparing models with and without each MLTC approach shown were all < 0.001. *N* = 495,465.

Abbreviations: BC, body system count; BI, Byles index; CC, condition count; CI, Charlson index; n/a, not applicable.

^a^ Multivariable model adjusted for age, sex, Townsend deprivation index, body mass index category, smoking status and alcohol use.

We repeated our Cox regression models separately for males and females, and the findings were similar (Table S1). When we stratified our findings by age group, we observed stronger associations between all four MLTC approaches and rates of emergency hospital admission (or death) in the 38–54 year age group compared with those aged 55–74 (Table S2). The values of Harrell's C‐statistic were similar between the two age groups.

Findings from sensitivity analyses suggested that results were very similar when using death as the sole outcome and when using updated versions of the CI (Table S3).

## Discussion

### Summary of findings

We compared four different simple approaches to characterising MLTC in the participants of the UK Biobank at the baseline assessment. We found variation between the approaches, with a higher prevalence of MLTC when using the two counts‐based approaches than the two indices. All four characterisations of MLTC showed strong associations with rates of emergency hospital admission (or death) over a 5‐year period, and this was particularly the case at younger ages. The approaches used had a moderate ability to predict individual risk of emergency hospital admission (or death).

### Interpretation of findings

We found a higher prevalence of MLTC using the count compared to the index approaches, similar to previous findings from analyses of primary care data [22, 23]. There was a high degree of overlap between the different approaches, with the less prevalent index approaches largely nested within the more prevalent count ones. The variation in prevalence is important, since it highlights how the choice of the list of conditions used to characterise individuals with MLTC needs careful consideration [5, 24].

We found that a greater MLTC score using any one of the four different approaches was associated with increased rates of emergency hospital admission or death. The one exception was the CI approach, where a score of 2 had a significantly lower hazard ratio than a score of 1 (Table 1). The majority of those with a score of 2 in the CI approach had a history of cancer and no other items (88.6%), compared with those with a score of 1, where the top three items were connective tissue disease (22.5%), myocardial infarct (21.4%) and cerebrovascular disease (16%). Participants were asked about cancer, which could have been diagnosed at any point in their lives [25], with cancers occurring further in the past assumed to be associated with a lower risk of emergency hospitalisation (or death).

Our finding of moderate predictive accuracy for emergency hospital admission using both the count and index approaches is similar to that seen in previous studies using population‐based cohorts [9, 22, 26]. Other work has highlighted how the use of variables beyond LTC can improve prediction of hospitalisation, for example, by the use of more detailed weightings and information on acute conditions in the adjusted morbidity groups tool [26], or blood test results and medications in the QAdmissions model [27]. Several studies have also incorporated different socioeconomic indicators based on a person's address, which, along with MLTC, are also predictors of hospital admission [26, 27, 28]. Finally, our finding of similar Harrell's C‐statistic for the prediction of emergency admission in younger and older age groups is also similar to that seen in previous studies [9, 26].

### Methodological considerations

Strengths of this study include that we used a single research cohort to compare a number of different approaches to the characterisation of MLTC, which to date has been mainly carried out in primary care datasets [9, 22, 26]. We also used an outcome that is ascertained from linkage to routine healthcare records and hence less susceptible to bias due to losses during follow‐up.

The UK Biobank Study is recognised to have a degree of healthy responder bias [29], as shown by the low prevalence of MLTC at baseline. This may in turn be a benefit for understanding trajectories of MLTC following this baseline assessment [30], along with the potential in the UK Biobank to investigate factors not typically available in routine data sources such as genotype and lifestyle risk factors. A related issue to highlight is that our assessment of MLTC was based on self‐report data, which were only collected if participants answered positively to an initial screening question (as outlined in the methods), and this may also have contributed to low prevalence of MLTC.

### Areas for future research

The implementation of ‘simpler’ approaches to characterise MLTC as used in this study is relevant to future research on MLTC for several reasons. First, these approaches have a key role to play in describing the burden of MLTC. This is important especially in hospital settings where the burden of MLTC is not yet well characterised and there is the potential for translation of MLTC research into impact by informing the development of health services that can meet the needs of growing numbers of people living with MLTC. Second, as there has been recent interest in the use of statistical clustering techniques to characterise MLTC [31], work using simpler approaches helps to highlight that the conditions used in the definition of MLTC, and their operationalisation within an individual dataset, can have a marked impact on the findings of subsequent cluster analysis [32]. Third, it may be that in some situations, such as patients receiving hospital care, the majority of individuals will not fall into clusters but rather have simpler patterns of MLTC [33]. Finally, many organ specialities are starting to explore interactions of individual conditions with multimorbidity (a comorbidity approach) and in this context, simpler approaches are useful [11].

## Conclusions

A range of simple approaches can be used to characterise MLTC. The two counts versus the two indices showed marked differences in the prevalence of MLTC in UK Biobank yet had similar performance in the prediction of emergency hospital admission. Approaches such as these are an important step in the characterisation of MLTC. They are being used in a rapidly growing number of research studies to improve understanding of the burden, causes and consequences of MLTC, with the overall aim to improve care for people living with MLTC.

## Conflicts of interest

None of the authors have conflicts of interest to declare.

## Supporting information


**Table S1**: Findings from Cox regression models for the relationship between each approach to characterising MLTC and rates of emergency hospital admission (or death), stratified by sex.
**Table S2**: Findings from Cox regression models for the relationship between each approach to characterising MLTC and rates of emergency hospital admission (or death), stratified by age group.
**Table S3**: Findings from Cox regression models for the relationship between the original Charlson index (as shown in Table 2) and two updated versions of the Charlson index[1, 2] and rates of emergency hospital admission (or death).

Supporting Information 2
