# Supplementary material for: Simple approaches to characterising multiple long‐term conditions (multimorbidity) and rates of emergency hospital admission: Findings from 495,465 UK Biobank participants
Source: J Intern Med. 2022 Sep 21;293(1):100–9. doi: 10.1111/joim.13567 (PMC10086957; doi:10.1111/joim.13567)
Supplement: Supplementary file 1 — Table S1: Findings from Cox regression models for the relationship between each approach to characterising MLTC and rates of emergency hospital admission (or death), stratified by sex. Table S2: Findings from Cox regression models for the relationship between each approach to characterising MLTC and rates of emergency hospital admission (or death), stratified by age group. Table S3: Findings from Cox regression models for the relationship between the original Charlson index (as shown in Table 2) and two updated versions of the Charlson index[1, 2] and rates of emergency hospital admission (or death). [file JOIM-293-100-s001.docx]

# Supporting Information

## Supporting Information – methods 1

### Further details of count and index measures used

#### Body system count (Dodds et al.)

1. Cardiovascular
2. Respiratory / ENT
3. Gastrointestinal / Abdominal
4. Renal / Urology
5. Endocrine / Diabetes
6. Neurology / Psychiatry
7. Musculoskeletal / Trauma
8. Haematology / Dermatology
9. Gynaecology / Breast
10. Immunological / Systemic disorders
11. Eye
12. Any history of cancer

[For all individual conditions within each of these 12 categories please see Supporting Information – methods 2.xls]

#### Count of conditions (Gallacher et al.)

1. Stroke/transient ischaemic attack (TIA)

2. Painful conditions

3. Hypertension

4. Depression

5. Asthma

6. Atrial fibrillation

7. Coronary heart disease

8. Dyspepsia

9. Diabetes

10. Thyroid disorders

11. Connective tissue disorders

12. Chronic obstructive pulmonary disease (COPD)

13. Anxiety

14. Irritable bowel syndrome

15. Alcohol problems

16. Other psychoactive substance abuse

17. Treated constipation

18. Chronic kidney disease

19. Diverticular disease

20. Peripheral vascular disease

21. Heart failure

22. Prostate disorders

23. Glaucoma

24. Epilepsy

25. Dementia

26. Schizophrenia/bipolar disorder

27. Psoriasis/eczema

28. Inflammatory bowel disease

29. Migraine

30. Chronic sinusitis

31. Anorexia or bulimia

32. Bronchiectasis

33. Parkinson’s disease

34. Multiple sclerosis

35. Viral hepatitis

36. Chronic liver disease

37. Osteoporosis

38. Chronic fatigue syndrome

39. Endometriosis

40. Meniere’s disease

41. Pernicious anaemia

42. Polycystic ovary

43. Cancer (any history)

[For all individual conditions within these 43 categories please see Supporting Information – methods 2.xls]

#### Charlson index

Score 1

- Myocardial infarct
- Congestive heart failure
- Peripheral vascular disease
- Cerebrovascular disease (TIA or CVA)
- Dementia
- Chronic pulmonary disease
- Connective tissue disease
- Ulcer disease
- Mild liver disease
- Diabetes (without end organ damage)

Score 2

- Hemiplegia*
- Moderate or severe renal disease
- Diabetes with end organ damage
- Any history of cancer (except leukaemia or lymphoma)
- Leukaemia
- Lymphoma

Score 3

- Moderate of severe liver disease

Score 6

- Metastatic solid tumour
- AIDS**

* Hemiplegia was not one of the long-term conditions that participants were able to self-report in UK Biobank and hence was not included.

** Participants were able to self-report “HIV/AIDS” as a long-term condition and we assumed that this related to HIV and not AIDS, and hence this part of the Charlson score was not used.

#### Byles index

Score 1

- Arthritis / rheumatism
- Angina
- Depression
- Fit / faint / funny turn
- Coronary artery bypass grafting
- Diabetes
- Stroke

Score 2

- Any history of cancer
- Liver disease

## Supporting Information - results

### Supporting Information Table 1: Findings from Cox regression models for the relationship between each approach to characterising MLTC and rates of emergency hospital admission (or death), stratified by sex

| Sex | Approach to characterising MLTC | | | | |
| --- | --- | --- | --- | --- | --- |
|  | None | CI | BI | CC | BC |
|  |  |  |  |  |  |
| Females (n=269,849) |  |  |  |  |  |
| Hazard Ratio (95% CI) |  |  |  |  |  |
| Score 0 | n/a | (ref) | (ref) | (ref) | (ref) |
| Score 1 | n/a | 2.03 (1.97-2.09) | 1.66 (1.62-1.70) | 1.43 (1.39-1.46) | 1.44 (1.40-1.49) |
| Score 2 | n/a | 1.57 (1.53-1.62) | 1.82 (1.77-1.87) | 1.96 (1.91-2.02) | 1.92 (1.87-1.98) |
| Score 3+ | n/a | 3.08 (2.91-3.26) | 2.64 (2.54-2.75) | 3.11 (3.03-3.20) | 2.78 (2.70-2.86) |
| C-statistic (95% CI) | 0.55 (0.550 – 0.555) | 0.586 (0.584 – 0.589) | 0.599 (0.596 – 0.601) | 0.623 (0.620 – 0.625) | 0.615 (0.612 – 0.617) |
|  |  |  |  |  |  |
| Males (n=225,616) |  |  |  |  |  |
| Hazard Ratio (95% CI) |  |  |  |  |  |
| Score 0 | n/a | (ref) | (ref) | (ref) | (ref) |
| Score 1 | n/a | 1.98 (1.93-2.03) | 1.64 (1.61-1.68) | 1.42 (1.39-1.46) | 1.44 (1.40-1.48) |
| Score 2 | n/a | 1.81 (1.76-1.87) | 2.00 (1.94-2.05) | 1.93 (1.88-1.98) | 1.89 (1.84-1.95) |
| Score 3+ | n/a | 3.15 (2.99-3.33) | 2.95 (2.83-3.07) | 3.11 (3.03-3.20) | 2.68 (2.60-2.76) |
| C-statistic (95% CI) | 0.571 (0.568 – 0.573) | 0.606 (0.603 – 0.609) | 0.613 (0.610 – 0.615) | 0.630 (0.627 – 0.632) | 0.620 (0.617 – 0.622) |
|  |  |  |  |  |  |

Cox regression models shown also adjusted for age band. *P-*values comparing models with and without each MLTC approach shown were all < 0.001. CI, Charlson index. BI, Byles index. CC, condition count. BC, body system count. n/a, not applicable.

### Supporting Information Table 2: Findings from Cox regression models for the relationship between each approach to characterising MLTC and rates of emergency hospital admission (or death), stratified by age group

| Age group | Approach to characterising MLTC | | | | |
| --- | --- | --- | --- | --- | --- |
|  | None | CI | BI | CC | BC |
|  |  |  |  |  |  |
| 38-54 years (n=191,449) |  |  |  |  |  |
| Hazard Ratio (95% CI) |  |  |  |  |  |
| Score 0 | n/a | (ref) | (ref) | (ref) | (ref) |
| Score 1 | n/a | 2.24 (2.14-2.33) | 1.94 (1.88-2.00) | 1.46 (1.42-1.51) | 1.46 (1.41-1.51) |
| Score 2 | n/a | 1.95 (1.87-2.03) | 2.26 (2.17-2.36) | 2.11 (2.04-2.19) | 2.00 (1.94-2.07) |
| Score 3+ | n/a | 4.40 (3.97-4.87) | 3.85 (3.59-4.14) | 3.68 (3.55-3.81) | 3.06 (2.96-3.17) |
| C-statistic (95% CI) | 0.523 (0.520 – 0.526) | 0.559 (0.556 – 0.563) | 0.584 (0.580 – 0.587) | 0.615 (0.611 – 0.618) | 0.611 (0.608 – 0.614) |
|  |  |  |  |  |  |
| 55-74 years (n=304,016) |  |  |  |  |  |
| Hazard Ratio (95% CI) |  |  |  |  |  |
| Score 0 | n/a | (ref) | (ref) | (ref) | (ref) |
| Score 1 | n/a | 1.93 (1.89-1.97) | 1.55 (1.52-1.58) | 1.38 (1.35-1.42) | 1.41 (1.37-1.45) |
| Score 2 | n/a | 1.60 (1.56-1.64) | 1.79 (1.75-1.83) | 1.84 (1.80-1.89) | 1.83 (1.78-1.88) |
| Score 3+ | n/a | 2.94 (2.82-3.07) | 2.60 (2.52-2.68) | 2.90 (2.83-2.97) | 2.58 (2.51-2.65) |
| C-statistic (95% CI) | 0.56 (0.553 – 0.557) | 0.594 (0.592 – 0.596) | 0.597 (0.595 – 0.600) | 0.619 (0.617 – 0.622) | 0.608 (0.605 – 0.610) |
|  |  |  |  |  |  |

Cox regression models shown also adjusted for sex and the sub-categories of age-band (e.g. 37-44 and 45-54 in the findings for the 37-54 year age group). *P-*values comparing models with and without each MLTC approach shown were all < 0.001. CI, Charlson index. BI, Byles index. CC, condition count. BC, body system count. n/a, not applicable.

### Supporting Information Table 3: Findings from Cox regression models for the relationship between the original Charlson index (as shown in Table 2) and two updated versions of the Charlson index[1, 2] and rates of emergency hospital admission (or death)

| Covariates in model | Approach to characterising MLTC | | | |
| --- | --- | --- | --- | --- |
|  | None | CI | Schneeweiss | Quan |
| Age and sex |  |  |  |  |
| Hazard Ratio (95% CI) |  |  |  |  |
| Score 0 | n/a | (ref) | (ref) | (ref) |
| Score 1 | n/a | 2.00 (1.96-2.04) | 2.19 (2.13-2.24) | 2.02 (1.97-2.08) |
| Score 2 | n/a | 1.67 (1.64-1.71) | 1.68 (1.65-1.71) | 1.52 (1.49-1.55) |
| Score 3+ | n/a | 3.12 (3.00-3.24) | 3.55 (3.42-3.69) | 2.87 (2.71-3.05) |
| C-statistic (95% CI) | 0.570 (0.569-0.572) | 0.601 (0.599 – 0.603) | 0.598 (0.596-0.600) | 0.589 (0.587-0.590) |
|  |  |  |  |  |
| Multivariable model* |  |  |  |  |
| Hazard Ratio (95% CI) |  |  |  |  |
| Score 0 | n/a | (ref) | (ref) | (ref) |
| Score 1 | n/a | 1.82 (1.78-1.85) | 1.96 (1.91-2.01) | 1.82 (1.77-1.87) |
| Score 2 | n/a | 1.64 (1.60-1.67) | 1.63 (1.60-1.66) | 1.51 (1.48-1.54) |
| Score 3+ | n/a | 2.75 (2.64-2.86) | 3.02 (2.90-3.14) | 2.56 (2.41-2.72) |
| C-statistic (95% CI) | 0.611 (0.609 – 0.613) | 0.629 (0.627 – 0.630) | 0.627 (0.626-0.629) | 0.621 (0.620-0.623) |

*Multivariable model adjusted for age, sex, Townsend deprivation index, BMI category, smoking and alcohol status.

*P-*values comparing models with and without each MLTC approach shown were all < 0.001. N = 495,465. CI, Charlson index. n/a, not applicable.

1. Schneeweiss S, Wang PS, Avorn J, Glynn RJ. Improved comorbidity adjustment for predicting mortality in Medicare populations. *Health Serv Res* 2003; **38**: 1103-20.

2. Quan H, Li B, Couris CM, Fushimi K, *et al*. Updating and validating the Charlson comorbidity index and score for risk adjustment in hospital discharge abstracts using data from 6 countries. *Am J Epidemiol* 2011; **173**: 676-82.
